# Como o COVID-19 pode Influenciar na Evolução de Pacientes com Síndromes Coronarianas Agudas?

**DOI:** 10.36660/abc.20230888

**Published:** 2024-03-06

**Authors:** Alexandre de Matos Soeiro

**Affiliations:** 1 Hospital das Clínicas da Faculdade de Medicina da Universidade de São Paulo Instituto do Coração São Paulo SP Brasil Instituto do Coração do Hospital das Clínicas da Faculdade de Medicina da Universidade de São Paulo, São Paulo, SP - Brasil; 2 Hospital Beneficência Portuguesa São Paulo SP Brasil Hospital Beneficência Portuguesa, São Paulo, SP - Brasil; 3 Hospital do Coração São Paulo SP Brasil Hospital do Coração, São Paulo, SP - Brasil

**Keywords:** COVID-19, Hospitalização, Infarto do Miocárdio com Supradesnivelameno do Segmento ST, Intervenção Coronária Percutânea/métodos, Infecções por Coronavírus, Embolia Pulmonar

A pandemia causada pelo vírus da COVID-19 mudou a forma como encaramos as doenças infecciosas. De uma forma mais intensa do que em outras situações, o COVID-19 esteve desde o início relacionado a maior número de eventos trombóticos, seja na sua apresentação aguda, com microtrombos pulmonares agravando o quadro de hipóxia, ou até mesmo na sua evolução tardia com grande número de eventos embólicos pulmonares. A interface entre endotélio e a cascata de coagulação apresentava-se modificada e diferentes mecanismos pró-trombóticos se mostravam de maneira desregulada, com expressão elevada do fator tecidual, CD40, moléculas de adesão leucocitárias, citocinas pró-inflamatórias, tromboxano e redução da produção de óxido nítrico. Junto aos pulmões, o coração é um dos órgãos mais afetados pela doença com acometimento global, seja miocárdico, arritmogênico ou coronariano.^1 - 5^

Dessa forma, o estudo apresentado de Baytugan et al.,^6^ traz uma descrição de pacientes com síndrome coronariana aguda com supradesnivelamento de ST (SCACSST) com COVID-19, comparando com o grupo sem COVID-19 em relação aos desfechos clínicos encontrados. Alguns pontos interessantes devem ser ressaltados, entre eles o fato da maior demora para os pacientes se apresentarem ao hospital. Isso foi muito comum durante a pandemia e com certeza agravou os desfechos relacionados à SCA. Observou-se também que pacientes com COVID-19 tinham idade mais avançada e mais comorbidades como diabetes e doença pulmonar obstrutiva crônica. Por último, de maneira incisiva e clara, os pacientes com COVID-19 evoluíram com piores desfechos como maior número de tromboses de stent, mais choque cardiogênico e maior mortalidade. Fato curioso foi a descrição de níveis mais elevados de troponina no grupo COVID-19. De forma mais simples, podemos relacionar isso ao maior atraso diagnóstico, porém vale ressaltar a possibilidade de agressão direta cardíaca causada pelo COVID-19, além da maior carga trombótica e inflamatória que a doença pode causar.^6^

Em 2020, Libby et al.,^7^ ressaltaram a interação entre disfunção endotelial e COVID-19. O autor levantava a hipótese de que o COVID-19 em sua essência fosse uma "doença endotelial". O COVID-19 em sua forma grave desencadeava uma tempestade de citocinas e desregulava mecanismos contra-regulatórios, afetando o balanço inflamatório, mecanismos de trombose e fibrinólise e a vasodilatação.^7^

De maneira semelhante ao estudo apresentado, uma das primeiras descrições sobre SCA e COVID-19 apresentava 18 pacientes, sendo desses apenas 8 com SCACSST e lesão coronariana trombótica. Já no início da pandemia, este estudo chamava a atenção mostrando uma taxa de 50% de mortalidade nesses pacientes e sinalizando altos níveis de D-dímero, achado incomum na fase aguda da SCA em outros estudos. Pela primeira vez, no contexto da SCA, se começava a levantar a hipótese de ruptura de placas, tempestade inflamatória, hipóxia, espasmos coronariano, microtrombos e disfunção endotelial como mecanismos conjuntos nessa forma de apresentação.^8^

Em 2020, Stefanini et al.^9^ descreveram 28 casos de SCACSST e COVID-19 na região da Lombardia na Itália. Em 24 pacientes, a SCACSST foi considerada a primeira manifestação do COVID-19. Semelhante a achados prévios, o autor chamava a atenção para uma mortalidade de 39,3%. Além disso, cerca de 40% não tinham doença obstrutiva coronariana no cateterismo.^9^

Seguindo a mesma linha, outro estudo publicado em 2020 comparou 348 casos de SCACSST e COVID-19 com 440 casos retrospectivos de 2019 sem COVID-19. Os autores mostraram menor tempo de ativação do serviço de saúde em 2019 vs. 2020 (75 vs. 87 min, p < 0,001), mas isso não impactou no tempo de revascularização da artéria culpada. No entanto, a mortalidade dos pacientes com COVID-19 foi de 21,7% vs. 9,3% (p = 0,012), mais uma vez mostrando a gravidade atrelada à infecção do COVID-19 na vigência da SCA.^10^

De maneira semelhante, outros estudos apresentaram dados sempre com desfechos piores no grupo de pacientes com COVID-19 e SCA. Porém, cada estudo traz suas particularidades e contribui para que tenhamos um melhor entendimento da doença e possamos melhorar o tratamento e os desfechos atrelados à mesma. Talvez a chave para melhores resultados seja realmente o contexto inflamatório, mas há muito ainda que precisa ser esclarecido e estudado.
