# Supplementary material for: Como o COVID-19 pode Influenciar na Evolução de Pacientes com Síndromes Coronarianas Agudas?
Source: Arq Bras Cardiol. 2024 Mar 6;121(1):e20230888. [Article in Portuguese] doi: 10.36660/abc.20230888 (PMC11081062; doi:10.36660/abc.20230888)
Supplement: Translation

# Translation: How Can COVID-19 Influence the Evolution of Patients with Acute Coronary Syndromes?

**Authors:** Alexandre de Matos Soeiro

**Affiliations:** 1 São Paulo SP Brazil Instituto do Coração do Hospital das Clínicas da Faculdade de Medicina da Universidade de São Paulo, São Paulo, SP - Brazil; 2 São Paulo SP Brazil Hospital Beneficência Portuguesa, São Paulo, SP - Brazil; 3 São Paulo SP Brazil Hospital do Coração, São Paulo, SP - Brazil

**Keywords:** COVID-19, Hospitalization, ST-Elevation Myocardial Infarction, Percutaneous Coronary Intervention/methods, Coronavirus Infections, Pulmonary Embolism

The pandemic caused by the COVID-19 virus has changed the way we view infectious diseases. In a more intense way than in other situations, COVID-19 was from the beginning related to a greater number of thrombotic events, whether in its acute presentation, with pulmonary microthrombi worsening the hypoxia, or even in its late evolution with a large number of pulmonary embolic events. The interface between the endothelium and the coagulation cascade was modified, and different pro-thrombotic mechanisms were deregulated, with elevated expression of tissue factor, CD40, leukocyte adhesion molecules, pro-inflammatory cytokines, thromboxane, and reduced production of nitric oxide. Along with the lungs, the heart is one of the organs most affected by the disease with global involvement, whether myocardial, arrhythmogenic, or coronary.^1 - 5^

Thus, the study presented by Baytugan et al.^6^describes patients with ST-elevation acute coronary syndrome (ST-ACS) with COVID-19, comparing them with the group without COVID-19 in relation to the clinical outcomes found. Some interesting points should be highlighted, including the fact that it takes longer for patients to present themselves to the hospital. This was very common during the pandemic and certainly worsened outcomes related to ACS. It was also observed that patients with COVID-19 were older and had more comorbidities, such as diabetes and chronic obstructive pulmonary disease. Finally, in an incisive and clear way, patients with COVID-19 developed worse outcomes, such as a greater number of stent thromboses, more cardiogenic shock, and higher mortality. A curious fact was the description of higher troponin levels in the COVID-19 group. More simply, we can relate this to the greater diagnostic delay; however, it is worth highlighting the possibility of direct cardiac aggression caused by COVID-19, in addition to the greater thrombotic and inflammatory load that the disease can cause.^6^

In 2020, Libby et al.^7^highlighted the interaction between endothelial dysfunction and COVID-19. The author raised the hypothesis that COVID-19, in essence, was an "endothelial disease". COVID-19, in its severe form, triggered a cytokine storm and deregulated counter-regulatory mechanisms, affecting the inflammatory balance, thrombosis and fibrinolysis mechanisms, and vasodilation.^7^

Similar to the study presented, one of the first descriptions of ACS and COVID-19 presented 18 patients, of which only 8 had ST-ACS and thrombotic coronary lesions. At the beginning of the pandemic, this study drew attention, showing a 50% mortality rate in these patients and signaling high levels of D-dimer, an uncommon finding in the acute phase of ACS in other studies. For the first time, in the context of ACS, the hypothesis of plaque rupture, inflammatory storm, hypoxia, coronary spasms, microthrombi, and endothelial dysfunction as joint mechanisms in this form of presentation was beginning to be raised.^8^

In 2020, Stefanini et al.^9^described 28 cases of ST-ACS and COVID-19 in the Lombardy region of Italy. In 24 patients, ST-ACS was considered the first manifestation of COVID-19. Similar to previous findings, the author drew attention to a mortality rate of 39.3%. Furthermore, around 40% did not have obstructive coronary disease at catheterization.^9^

Following the same line, another study published in 2020 compared 348 cases of ST-ACS and COVID-19 with 440 retrospective cases from 2019 without COVID-19. The authors showed a shorter health service activation time in 2019 vs. 2020 (75 vs. 87 min, p < 0.001), but this did not impact the revascularization time of the culprit artery. However, the mortality of patients with COVID-19 was 21.7% vs. 9.3% (p = 0.012), once again showing the severity linked to COVID-19 infection during ACS.^10^

Similarly, other studies presented data always with worse outcomes in the group of patients with COVID-19 and ACS. However, each study brings its own particularities and helps us to have a better understanding of the disease and to improve the treatment and outcomes linked to it. Perhaps the key to better results really is the inflammatory context, but there is still a lot that needs to be clarified and studied.
